# Correlating clinical course with subcortical shape in provisional tic disorder

**DOI:** 10.1017/S1092852924002190

**Published:** 2024-11-28

**Authors:** Tiffanie Che, Soyoung Kim, Deanna J. Greene, Ashley Heywood, Jimin Ding, Tamara Hershey, Bradley L. Schlaggar, Kevin J. Black, Lei Wang

**Affiliations:** 1 Department of Psychiatry and Behavioral Health, Ohio State University Wexner Medical Center, Columbus, OH, USA;; 2 Departments of Psychiatry and Radiology, Washington University in St. Louis School of Medicine, St. Louis, MO, USA;; 3 Department of Cognitive Science, University of California San Diego, La Jolla, CA, USA;; 4 Department of Psychiatry and Behavioral Sciences, Northwestern University Feinberg School of Medicine, Chicago, IL, USA;; 5 Department of Mathematics and Statistics; Department of Medicine, Washington University in St. Louis, St. Louis, MO, USA;; 6 Departments of Psychiatry, Neurology, Psychological and Brain Sciences and Radiology, Washington University in St. Louis School of Medicine, St. Louis, MO, USA;; 7 Kennedy Krieger Institute, Baltimore, MD, USA;; 8 Departments of Neurology and Pediatrics, Johns Hopkins University School of Medicine, Baltimore, MD, USA; 9 Departments of Psychiatry, Neurology, Radiology and Neuroscience, Washington University in St. Louis School of Medicine, St. Louis, MO, USA

**Keywords:** Tic disorders, neuroanatomy, neurodevelopmental disorders, FreeSurfer, subcortical shape, tourette syndrome, structural MRI

## Abstract

**Objective.:**

This study examined children at the onset of tic disorder (tics for less than 9 months: NT group), a population on which little research exists. Here, we investigate relationships between the baseline shape and volume of subcortical nuclei, diagnosis, and tic symptom outcomes.

**Methods.:**

187 children were assessed at baseline and a 12-month follow-up: 88 with NT, 60 tic-free healthy controls (HC), and 39 with chronic tic disorder/Tourette syndrome (TS), using T1-weighted MRI and total tic scores (TTS) from the Yale Global Tic Severity Scale to evaluate symptom change. Subcortical surface maps were generated using FreeSurfer-initialized large deformation diffeomorphic metric mapping. Linear regression models correlated baseline structural shapes with follow-up TTS while accounting for covariates, with relationships mapped onto structure surfaces.

**Results.:**

We found that the NT group had a larger right hippocampus compared to HC. Surface maps illustrate distinct patterns of inward deformation in the putamen and outward deformation in the thalamus for NT compared to controls. We also found patterns of outward deformation in almost all studied structures when comparing the TS group to controls. The NT group also showed consistent outward deformation compared to TS in the caudate, accumbens, putamen, and thalamus. Subsequent analyses including clinical symptoms revealed that a larger pallidum and thalamus at baseline correlated with less improvement of tic symptoms at follow-up.

**Conclusion.:**

These observations constitute some of the first prognostic biomarkers for tic disorders and suggest that these subregional shape and volume differences may be associated with the outcome of tic disorders.

## Introduction

Persistent (chronic) tic disorders (CTD) were once thought to be rare but are now known to affect a substantial number of elementary school children.^1^ Tics are sudden, repetitive, non-rhythmic movements or vocalizations such as blinks or grunting.^2^ Tics affect at least 20% of children, though only about 3% of all children have tics for a full year, the requirement to diagnose a chronic tic disorder or Tourette syndrome (CTD/TS).^3, 4^ When tics are present but less than a year has passed since the first lifetime tic, Provisional Tic Disorder is diagnosed.^1^ Efforts to identify biomarkers for tics and study the pathophysiology behind tic disorders have recently been increasing, although our understanding is still limited.^5, 6^

A paucity of available autopsy data elevates the importance of *in vivo* neuroimaging in studying the pathophysiology of tic disorders. Studies exploring differences in subcortical structure and function have often been contradictory, with some finding no significant differences in basal ganglia volumes or shape between children with TS and matched control children.^6^ Two groups found greater putamen volume in TS compared to HC (13 TS and 16 HC; 14 TS and 15 HC), but a larger study (49 TS and 42 HC) found smaller volume.^7–9^ A large study of basal ganglia volume (154 TS and 130 HC) found the caudate to be 4.9% smaller in the TS group compared to tic-free subjects.^10^ Smaller studies (14 TS and 14 HC; 18 TS and 12 HC) also found lower caudate volume,^11, 12^ but another large pediatric study (103 TS and 103 HC) identified no difference between TS and control groups.^13^

Previous such studies have also focused only on TS (diagnosed chronic tics) and control samples. Therefore, we cannot determine whether the identified differences reflect an underlying cause of tics or secondary changes due to prolonged tic presence. Examining children at the onset of tic symptoms will more likely lead to identifying biomarkers related to the primary cause of tics.

The ongoing NewTics study examined children who had tics for less than 9 months (new tics, or NT).^14, 15^ Previously, little research existed on this population, with even fewer results on prognosis, and those had been contradictory.^4^ The NewTics study tested whether features of subcortical structures measured shortly after tic onset could predict symptom severity 12 months after tic onset. A previous volumetric MRI analysis using data from 65 children with NT (a subset of the current sample) found that striatal volumes did not predict outcome, but a larger hippocampus at baseline predicted worse severity at follow-up.^16^ However, using whole-structure volume estimates alone may yield false negative findings by overlooking local deformities in shape.

In the present study, we further investigated neurobiological characteristics and predictors of tic disorders by examining relationships of the *shape* of these subcortical structures with tic symptom outcomes (NT) and with diagnosis (NT, TS, and tic-free controls). Three-dimensional surface analysis can detect subtler or more localized volumetric changes that are not revealed in whole-structure, scalar volumetric analysis.^17^ Previously, diffeomorphic mapping of structural magnetic resonance imaging (MRI) has successfully mapped pathological biomarker patterns onto surface-based representations of anatomical structures.^18^ We also take advantage of a much larger data set, a superset of the sample previously studied with scalar volumetrics.^16^

We hypothesized that baseline volumes would differ across NT, TS, and control groups and that subcortical shape would demonstrate distinct patterns of shape deformation in tic disorders. We further predicted that we would find distinct regions of shape deformation in subcortical structures at baseline that predict clinical outcome in terms of tic severity changes 3–12 months later. We also tested whether shape deformation analyses would confirm the previous finding that hippocampal volume predicted symptom severity outcome using a 3D method in an expanded sample.

## Methods

### Subjects and data collection

#### Subjects.

The sample consisted of 187 children (135 M, 52 F) across 3 groups: children examined within 9 months after tic onset (median 3.5 months; new tic, or NT), tic-free children with no parental or sibling history of tics (healthy controls, HC), and children who at the time of screening already have TS/CTD (TS group).^14^ Every participant in the TS group was diagnosed by a TS specialist (KJB or BLS). Most were diagnosed based on DSM-IV-TR criteria, which are slightly more stringent than the DSM-5 criteria. NT children returned for clinical evaluation at the one-year anniversary of the best estimate of tic onset. For TS and HC children, follow-up visits occurred as near as possible to the same time after screening as it did for their matched NT child, based on age, sex, and handedness. Healthy controls were collected across different studies/sources. Detailed information about recruitment methods appeared elsewhere.^14^ Each child assented, and a parent/guardian gave informed written consent. Data from other projects were shared after appropriate human subjects’ review and consent. The authors assert that all procedures contributing to this work comply with the ethical standards of the relevant national and institutional committees on human experimentation and with the Helsinki Declaration of 1975, as revised in 2008. All procedures involving human subjects/patients were approved by the Washington University Human Research Protection Office (IRB, protocols 201 109 157 and 201 707 059).

#### Enrollment criteria.

Participants were ages 5–15 at enrollment (all but 3 ages 5–10). NT children had current tic symptoms, with the first tic starting less than 9 months before enrollment. Exclusion criteria are detailed elsewhere.^14^ The TS group included children who met DSM-5 criteria for TS/CTD at enrollment. The exclusion criteria used reflect those of the NT group. Control children were confirmed to have no tics after a thorough history from the child and parent separately, neurological examination, and at least 10 minutes of observation via video while the child sat alone.

#### Clinical data collection.

For participants exhibiting tic symptoms, a best-estimate date of onset was recorded after careful inquiry as described in.^19^ Total tic scores from the Yale Global Tic Severity Scale (YGTSS), reflecting current tic severity, were determined during a neurological and psychiatric examination performed by author KJB, supplemented with remote video observation of the participant sitting alone. Additional assessments, including a K-SADS diagnostic interview and measures of OCD and ADHD symptoms, were done at the time of screening.^14^ The Total Tic Score (TTS) comprises half of the YGTSS score and has a range of 0–50; a higher score indicates more severe tic symptoms.^20^

#### Imaging data collection.

All subjects at entry were scanned with T1-weighted-magnetization prepared rapid gradient echo (MPRAGE) and T2-weighted sequences using three different 3 T scanners across the data acquisition period. Details of scan parameters are given in Kim et al. (2020).^16^ A portion (107/187) of the scans (the newer ones) were acquired with a prospective motion correction sequence (vNavs).^21^

Additional measures were taken prior to scanning to reduce motion effects on images, including training in a mock scanner, an informational video, and a game for children to practice holding still during scanning, adapted from a previous study in children.^22^

### Data processing/image processing

Data processing began with the use of the FreeSurfer (version 6.0.0) software’s probabilistic voxel-based classification, which provided initial subcortical segmentations.^23^ Surfaces of the hippocampus, amygdala, basal ganglia (caudate, putamen, pallidum, and nucleus accumbens), and thalamus were automatically generated for each participant using multi-atlas. FreeSurfer-initialized large deformation diffeomorphic metric mapping (FS + LDDMM), which utilizes automated brain segmentations based on multiple template images and allows for image alignment and intensity normalization to produce smooth transformations for each region of interest.^23, 24^ Combining maps from multiple atlases that best match an individual’s scan features has shown improved segmentation accuracy and reduced biases.^25^ An experienced rater (the first author), while blind to diagnosis, inspected the final surfaces and made minor manual edits to the initial segmentation on poor surface maps of 6 subjects. The edited segmentations were then visually verified by another author (LW) and then reprocessed via LDDMM to yield accurate maps to be included in subsequent surface analyses.

To equalize the effect of total brain volume across participants, surface deformation was then scaled with a scale factor calculated for each subject using the population total intracranial volume (TIV), the individual’s TIV, and the voxel size of their scan. To calculate the (one-dimensional) scale factor, we used the formula: (Population TIV/Individual TIV)^⅓^ × Voxel Size.

Local shape variation for each participant was calculated from the population average of all participants by quantifying the vertex-to-vertex perpendicular change between surfaces, which were assigned a positive (outward variation from the population average) or negative (inward variation from the population average) value.^23^ Subcortical volumes for each participant were determined using the volume enclosed within the surfaces. FreeSurfer also reported estimated total intracranial volume (TIV) to be used as a covariate in surface analyses, as TIV estimated by FreeSurfer segmentation is correlated with subcortical volumes.^24^

### Statistical analysis

#### TTS.

Paired T-tests were conducted to determine the significance of TTS changes from baseline to 12 months within the NT group.

#### TIV.

We first conducted a one-way ANOVA on TIV (dependent variable) to determine group (independent variable) effects with post hoc Tukey HSD tests for pairwise differences, with and without age and sex as covariates. The tests showed significant group effects (see results); therefore, TIV was used to scale the surfaces.

#### Scanner type.

We first conducted a chi-square test to determine whether scanner types differed between groups. Additionally, we conducted one-way ANOVA tests to determine the effects of scanner type on structural volumes. We found that the subcortical structures did not differ in volume based on the scanner type, and subsequent ANCOVA and post hoc tests found that group differences in TIV between NT and healthy controls persisted after controlling for the scanner type. Thus, we did not use the scanner type as a covariate in subsequent analyses.

#### Race.

The same tests done for scanner type (see above) were applied to participant race as well, and similar results were found. We thus also did not use race as a covariate in subsequent analyses.

#### Subcortical volumes.

Using the surface-defined volumes for each subcortical structure, we first found estimated marginal means and standard errors for each group. ANCOVA and post hoc tests on subcortical volumes (dependent variables) were then conducted to compare group (independent variable) differences of baseline structural volumes, using age and sex as covariates. All structures were examined by taking the sum of left and right subcortical structural volumes, since we did not have a hypothesis regarding laterality. Further analyses were conducted to examine left and right structures separately. We also examined hippocampal volume group differences in a subset of subjects who had vNavs. Analyses run in R used version 4.0.5, with psych and ggplot2 packages.^26, 27^ In order to control for the issue of multiple comparisons, we also applied the Bonferroni adjustment (for seven structures when looking at whole structures and fourteen structures when examining left and right structures separately), setting a more stringent significance threshold for each individual test within the analysis. We additionally employed Cohen’s d as a standardized measure to quantify the effect size of the observed differences in structural volumes between groups.

We then performed a partial correlation analysis using baseline structural volume to predict 12-month TTS in the NT group while controlling for TTS at screening, age, and sex.

#### Shape.

For group comparisons, ANCOVAs were conducted to compare pairwise group differences of baseline surface shape (i.e., NT versus HC, TS versus HC, and TS versus NT). All models included covariates for age and sex. Surface comparisons were conducted using SurfStat implemented in MATLAB.^28^ This software applies random field theory (RFT) to identify significant clusters of vertices at the family-wise error rate (FWER) of p < 0.05 within each subcortical structure to account for the multiple comparisons inherent in surface maps.^29^ Group differences were visualized as a color map displayed on the overall average surface.^23^

In order to concentrate on the prominent regions found in the TS-HC surface comparison, we then extracted the significant vertices found in this comparison in applicable structures and found the mean values for each subject of the deformation from the population average at these vertices. Additionally, significant vertices from the NT-HC surface comparisons were studied with partial correlation analyses on each subject’s mean deformation value and their 12-month clinical score (TTS), while controlling for TTS at screening, age, and sex. Subsequently, we repeated this process using significant vertices we extracted from the NT-TS surface comparisons.

## Results

### Subject demographics and clinical features

In total, we enrolled 127 NT participants. Of these, 88 NT participants had an initial scan and were included in our analyses, in addition to 60 HC and 39 TS participants (Table 1).

### Baseline TIV

A one-way ANOVA on TIV with age and sex as covariates revealed significant group differences [F(2,182) = 3.316, p = 0.004]. Post hoc Tukey HSD tests revealed that the NT group had a smaller TIV than controls at baseline (NT = 1484 ± 154 cm^3^ and HC = 1596 ± 141 cm^3^; p = 0.03). Group differences in TIV between NT and healthy controls remained significant after controlling for both scanner type and race independently. Additionally, the TIV differed significantly across scanner types (p = 0.04) and race (p = 0.04). Since TIV differed significantly among groups, we included TIV as a factor when scaling the surfaces.

### Baseline group comparisons of subcortical structural volumes

#### Hippocampus.

Group differences for whole hippocampal volume nearly reached significance at p = 0.06 [F(2,182) = 2.81]. The difference reached statistical significance for the right hippocampus (p = 0.04). NT participants had on average an 8.5% larger right hippocampus compared to the children without tics (Table 2). As this difference did not survive after Bonferroni adjustments, we interpret these results with caution.

#### Amygdala, caudate, accumbens, putamen, pallidum, and thalamus.

No group differences were found.

### Correlation of 12-month TTS with baseline structural volume

Longitudinal TTS analyses included 80 NT subjects. Over the course of 3 to 12 (median 8.5) months between the baseline and second visits, the average TTS decreased significantly from 16.96 ± 5.64 to 14.16 ± 6.94 (t = 3.82, df = 79, p = 0.0003).

#### Hippocampus, amygdala, accumbens, putamen, and TIV.

Scaled baseline structural volumes and TIV did not significantly predict TTS changes in the NT group. All correlations had r between −0.14 and 0.24 (Table 3).

#### Caudate.

**Figure 1.**
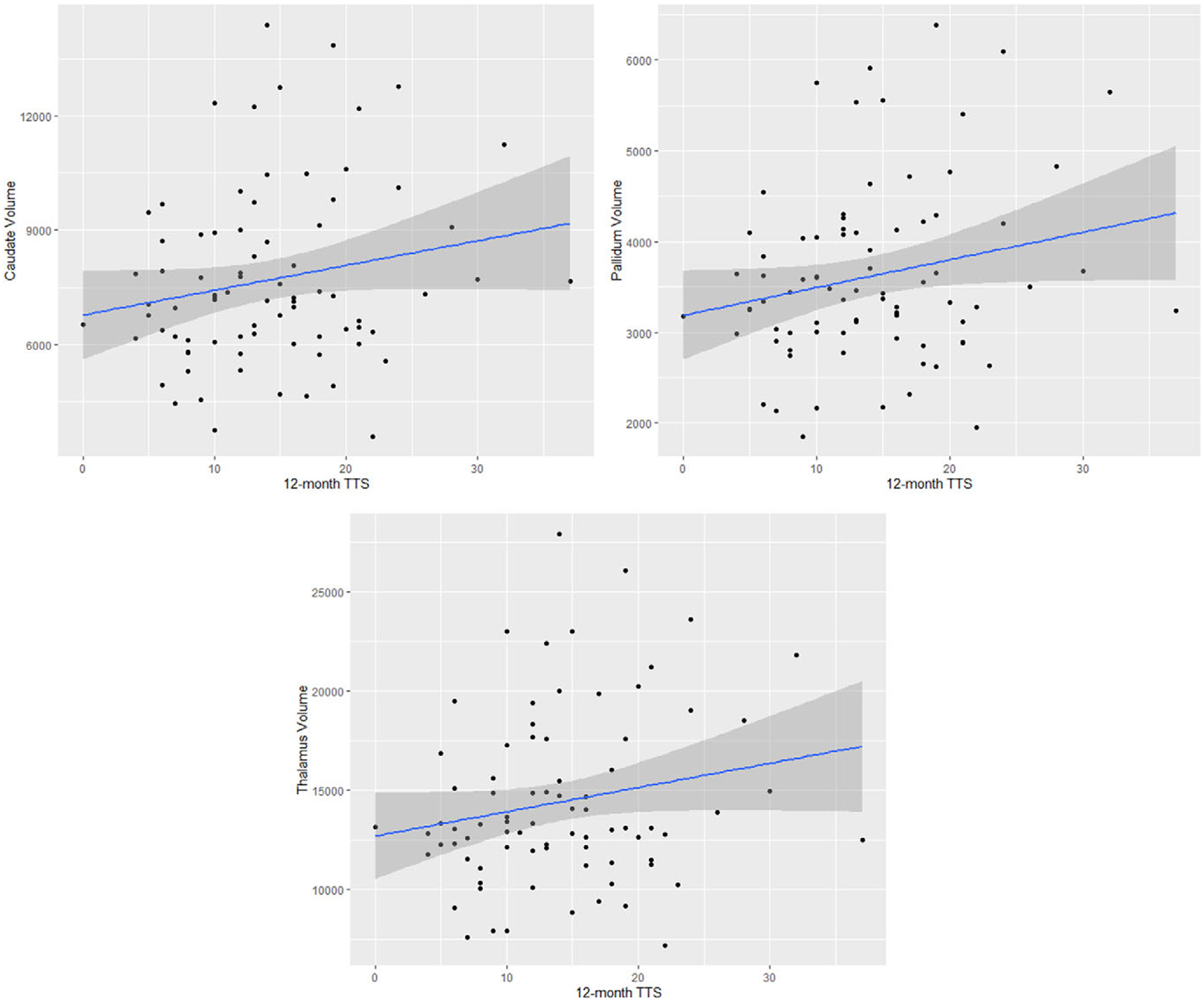
12-month TTS scores & structural volumes. Scatterplots showing the relationships between 12-month TTS scores and structural volumes.

We found a positive correlation between baseline caudate volume and TTS change from baseline to 12-month measurements that was near significance (r = 0.21, p = 0.06); that is, those with a larger caudate at baseline tended to show less subsequent improvement in tic symptoms (Table 3, Figure 1).

#### Pallidum.

We found a significant positive correlation between baseline pallidal volume and TTS change from baseline to 12-month measurements (r = 0.24, p = 0.04). A larger pallidum at baseline was significantly correlated with less improvement of tic symptoms (Table 3, Figure 1).

#### Thalamus.

We found a significant positive correlation between baseline thalamic volume and TTS change from baseline to 12-month measurements (r = 0.23, p = 0.05). A larger thalamus at baseline was significantly correlated with less improvement of tic symptoms (Table 3, Figure 1).

### Shape comparison between NewTics and Tourette syndrome versus healthy controls

#### Putamen.

**Figure 2.**
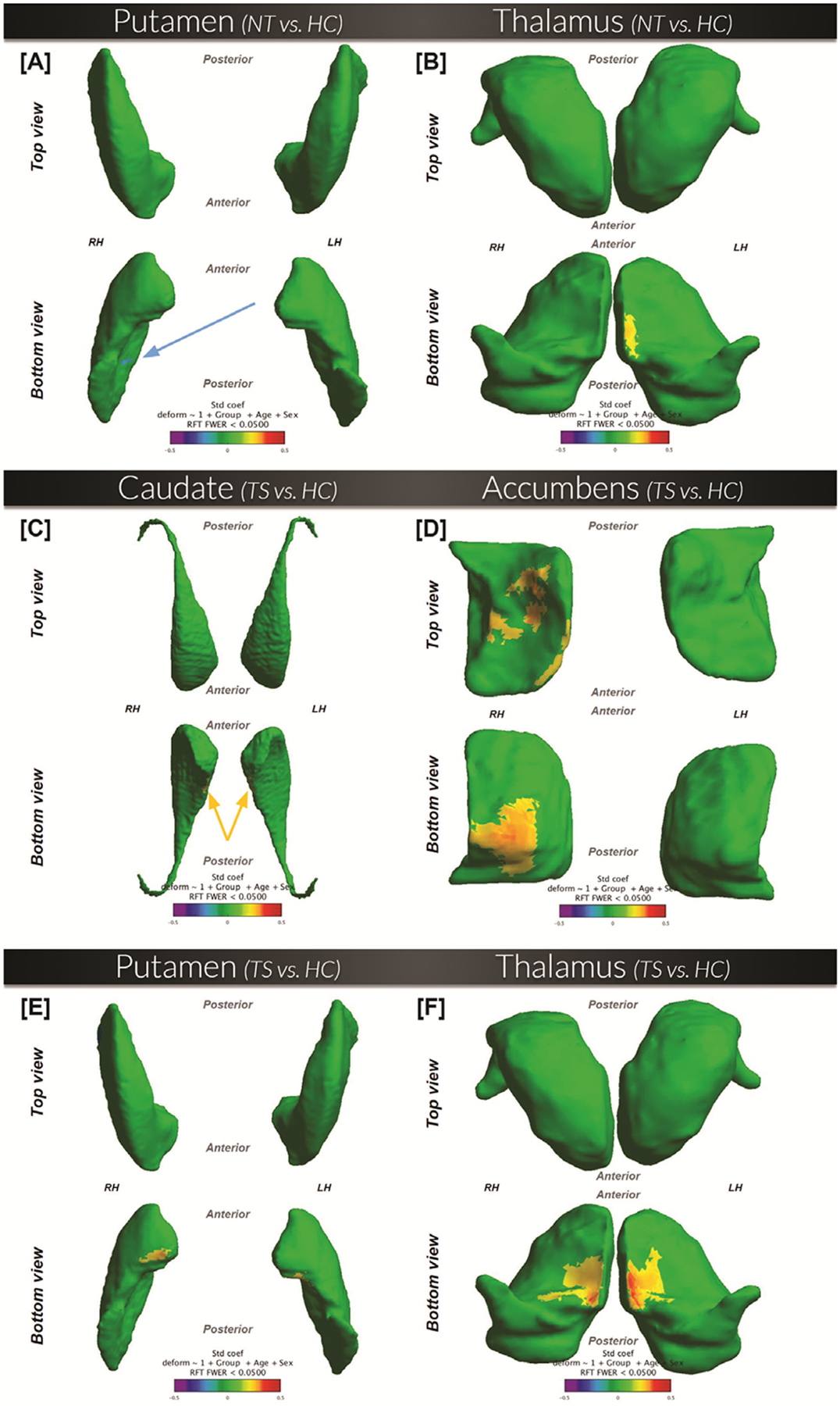
NewTics versus healthy controls (n = 148; 88 NT and 60 HC) and Tourette syndrome versus healthy controls (n = 99; 29 TS and 60 HC). Shape comparison between specified groups, while controlling for the effects of age and sex. Surfaces are scaled by total intracranial volume and voxel resolution. Cooler shades represent a greater inward deformity of the first group relative to the second, whereas warmer shades represent greater outward deformity. RFT = comparisons that passed the random field theory threshold.

(Figure 2 Panel A). Inferiorly, inward deformation (**NT** < HC) is present in the right medial putamen.

#### Thalamus.

(Figure 2 Panel B). Outward deformation (**NT** > HC) is present in the medial aspect of the inferior left thalamus.

#### Caudate.

(Figure 2 Panel C). Inferiorly, regions of outward deformation (**TS** > HC) are seen in the left and right medial caudate.

#### Accumbens.

(Figure 2 Panel D). Superiorly and inferiorly, outward deformation (**TS** > HC) is present in some regions of the right accumbens.

#### Putamen.

(Figure 2 Panel E). Inferiorly, outward deformation (**TS** > HC) is present in small regions of the left and right medial anterior putamen.

#### Thalamus.

(Figure 2 Panel F). Outward deformation (**TS** > HC) is present towards the medial ends of the left and right thalamus.

After extracting the significant vertices found in the TS-HC surface comparison in the caudate, accumbens, putamen, and thalamus, we calculated the mean values for each subject of the deformation referenced to the population average surface at these vertices (Table 4). We then performed an ANCOVA for each structure to test for group differences among NT, HC, and TS while controlling for age and sex.

#### Caudate.

We found inward deformation in both the HC and NT groups compared to the TS group (Table 4).

#### Accumbens, putamen, thalamus, and all significant vertices as a whole:

there were no statistically significant differences between groups.

### Shape comparison between NewTics versus Tourette syndrome

#### Hippocampus.

**Figure 3.**
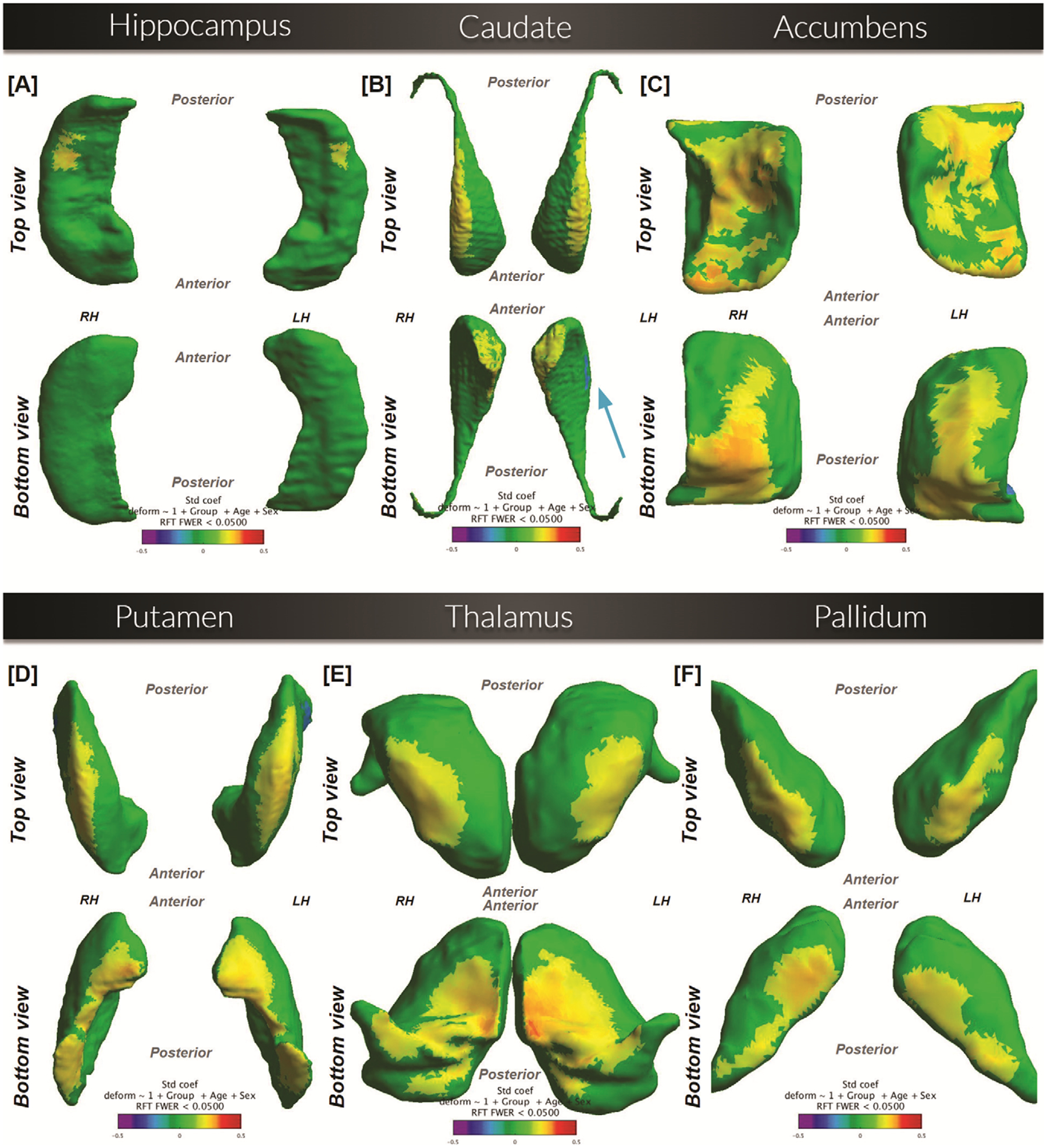
Shape comparison between NewTics versus Tourette syndrome (n = 127; 88 NT and 39 TS), while controlling for the effects of age and sex. Surfaces are scaled by total intracranial volume and voxel resolution. Cooler shades represent a greater inward deformation of the NT group relative to the TS group, whereas warmer shades represent greater outward deformation. RFT = comparisons that passed the random field theory threshold.

(Figure 3 Panel A). Superiorly, outward deformation (NT > TS) is present, concentrated towards the lateral-posterior parts of both the left and right hippocampus.

#### Caudate.

(Figure 3 Panel B). Superiorly, outward deformation (NT > TS) is present along the lateral edge of both the left and right caudate. Inferiorly, NT > TS in the medial anterior parts of the left and right caudate. Additionally, a small region of inward deformation (NT < TS) exists inferiorly along the lateral edge of the left caudate.

#### Accumbens.

(Figure 3 Panel C). Outward deformation (NT > TS) is present in large regions of both the left and right hemispheres superiorly and inferiorly.

#### Putamen.

(Figure 3 Panel D). Outward deformation (NT > TS) is present in large regions of both the left and right hemispheres superiorly and inferiorly.

#### Thalamus.

(Figure 3 Panel E). Superiorly, outward deformation (NT > TS) is present in large regions of both hemispheres. Similar patterns are concentrated towards the medial side of both the left and right thalamus inferiorly.

#### Pallidum.

(Figure 3 Panel F). Outward deformation (NT > TS) is present in both the left and right pallidum inferiorly and superiorly.

### Correlation of 12-month TTS with baseline structural shape

When comparing the NT group with healthy controls, we found that 18 out of the 13 638 vertices on the putamen surface had a significant deformation value. On the thalamus, 45 out of 9580 vertices were significant.

Next, we tested whether these focal deformations at baseline predicted clinical change at follow-up while controlling for screen TTS, age, and sex. We did not find a significant correlation between mean deformation values and 12-month TTS (putamen: r = 0.02, p = 0.86; thalamus: r = 0.11, p = 0.34).

When comparing the NT group with the TS group, numbers of significant vertices on structure surfaces were as follows: 183/13222 on the hippocampus surface; 3734/24744 on the caudate surface; 2279/5804 on the accumbens surface; 1030/5398 on the pallidum surface; 3345/13638 on the putamen surface; and 1956/9580 on the thalamus surface. Using these vertices, we found the correlation and p-values between each NT subject’s mean deformation value in the significant vertices of the NT-TS comparison and their 12-month TTS, while controlling for screen TTS, age, and sex (hippocampus: r = −0.10, p = 0.37; caudate: r = −0.13, p = 0.27; accumbens: r = −0.09, p = 0.42; pallidum: r = −0.14, p = 0.24; putamen: r = −0.14, p = 0.21; thalamus: r = −0.13, p = 0.24). None of these reached statistical significance.

## Discussion

In this study, we focus on the first year of tic development (NT group) and identified several baseline subcortical volume and shape characteristics related to baseline tic symptoms and some that predict clinical tic outcome 3 to 12 (median 8.5) months later.

Baseline volume analyses showed that the right hippocampus was on average 8.5% larger in NT children compared to healthy controls. A larger pallidum or thalamus at baseline predicted weaker improvement of tic symptoms at follow-up. Surface analyses demonstrated distinct patterns of subcortical surface deformation in several structures across all group comparisons.

In the significant vertices from the caudate TS-HC surface comparison, the TS group showed an overall trend of greater outward deformation from the population average surface compared to both the HC and NT groups. Since the NT group has had tics only for a few months, we can rule out the possibility that these subcortical volume differences are caused by living with tics for years; they are more likely related to the cause of tics.

### Functional implications

#### Hippocampus.

**Figure 4.**
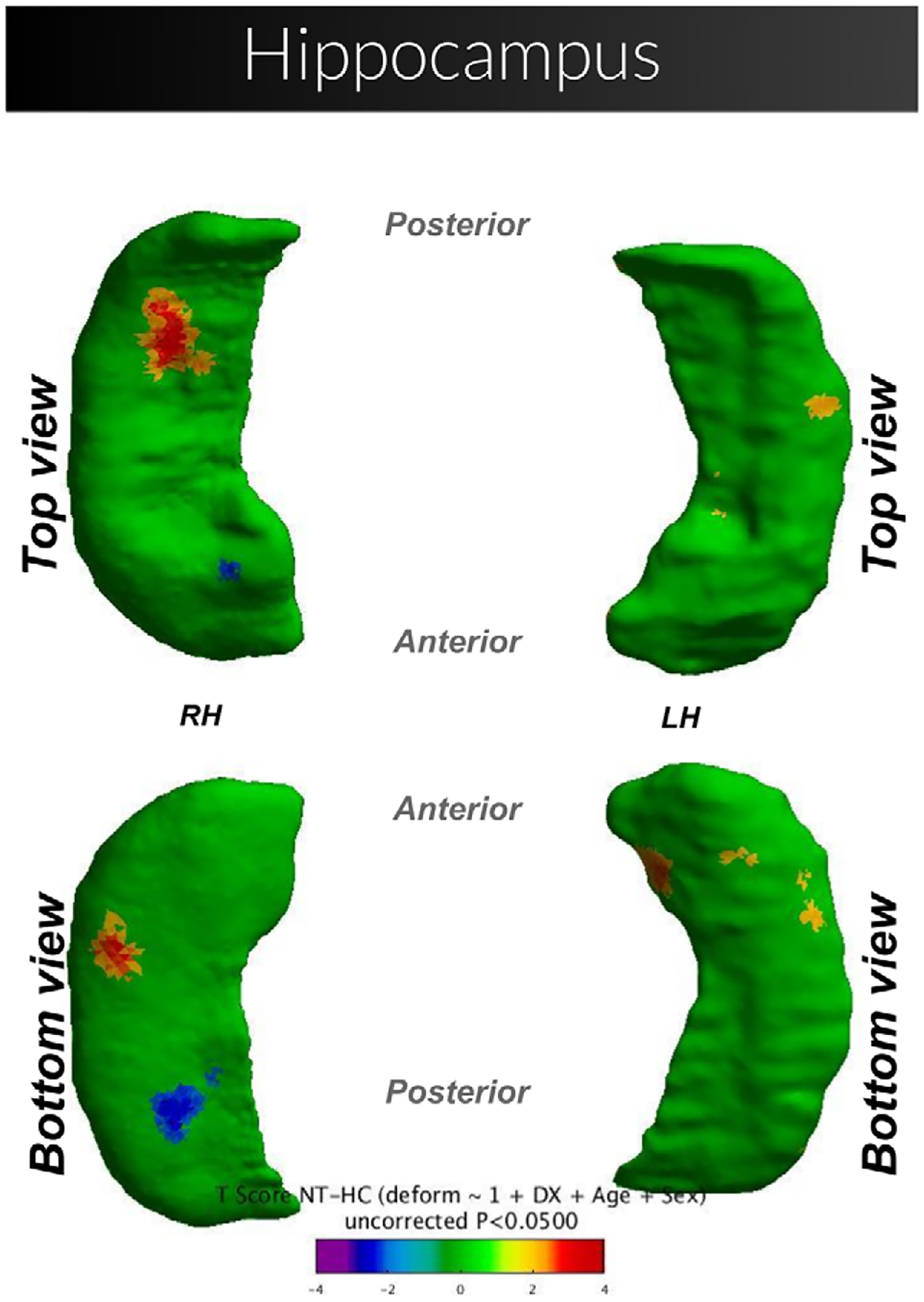
NewTics versus healthy controls in vNavs subjects (n = 76; 56 NT and 20 HC). Shape comparison between specified groups, while controlling for the effects of age and sex. Surfaces are scaled by total intracranial volume and voxel resolution. Cooler shades represent a greater inward deformity of the NT group relative to controls, whereas warmer shades represent greater outward deformity.

Children in the NT group had larger right hippocampal volume compared to healthy controls. The NT group also exhibited localized volume increases compared to the TS group. The hippocampus plays a role in memory consolidation in both the cognitive and motor domains.^30^ Evidence is strong for a relationship with visuospatial memory, with a larger hippocampus associated with higher performance and traumatic injury to the right hippocampus correlated to lower memory performance.^31, 32^ Our conjecture is that enlargement of the hippocampus may lead to abnormally strong preservation of motor memory. In children with TS, those with more persistent visuomotor memory show more severe tics and take longer to unlearn a previously learned motor pattern.^33^ As the NT group had a larger right hippocampus at baseline compared to healthy controls, we hypothesize that this region may be implicated in tic development and/or persistence. Previous work has found a larger hippocampus in children with *chronic* tics (TS),^34^ but the current results, confirming our results with a smaller sample and a different method,^16^ show that the larger hippocampus is present very early in the course of tic disorder and cannot result from adaptation to years of ticcing. This conclusion is consistent with a study of almost 5000 people with TS, which associated genetic risk variants for TS with gene variants linked to a larger hippocampus.^35^ Kim et al, using a subset of our sample, additionally found that a larger hippocampus at baseline predicted worse severity at follow-up, though we did not find a similar pattern in this analysis. One potential explanation for this inconsistency is that the present larger data set included more scans that did not use a prospective motion correction sequence (vNavs). In our analysis of the subset of subjects who had vNavs (56 NT, 31 TS, 20 HC), we found that the NT group had a numerically larger hippocampus than controls at baseline (Table 2). While the group difference did not reach statistical significance (p = 0.10), the effect sizes were similar to Kim et al. (r = 0.248 and r = 0.220, respectively). We conclude that the current difference is comparable to that reported by Kim et al. We further performed an accompanying hippocampal surface shape analysis in these subjects, shown in Figure 4.

#### Basal Ganglia (accumbens, pallidum, putamen, and caudate).

A larger baseline pallidum predicted a weaker improvement of tic symptoms. When analyzing shape differences, we found significant patterns of localized volume increases in NT children compared to TS children in all four structures. However, the putamen in the NT group exhibited localized volume loss when compared to healthy controls, but this deformation was concentrated towards the posterior end. The TS group exhibited localized volume increases in the caudate, accumbens, and putamen when compared to healthy controls. In the significant vertices from the TS-HC surface comparison of the caudate, we found an overall trend of greater outward deformation (compared to the population average) in the TS group compared to both the HC and NT groups.

We had hypothesized that smaller caudate volume would predict a worse outcome (higher TTS scores at 12 months) based on previous findings.^10, 14^ For example, a smaller caudate in 43 children with TS was found to predict more severe tic symptoms an average of 7.5 years later.^36^ However, in the NT group, we found the opposite at p = 0.06 (Table 3). Similarly, we had hypothesized that caudate volume would be significantly lower in NT when compared to healthy controls, though our findings suggest that caudate volume in NT was similar or slightly higher compared to controls (Table 2). Our contrasting results could be a result of exploring a newly studied population (our NT group) rather than only children with established TS in previous studies. Alternatively, the smaller caudate in the older work in TS^10^ may have been an artifact attributable to greater head movement and more parsimoniously attributed to more head movement in those with tics at the time of the scan. The present work has the advantage of prospective movement correction in a meaningful fraction of participants, minimizing such confounding.

The basal ganglia are subcortical nuclei that, along with their associated connections, have been a large focus of TS/CTD research. They are involved in motor control in other movement disorders such as Parkinson’s and Huntington’s disease. Cortico-striatal-thalamo-cortical (CSTC) circuits are involved in inhibitory control and habit formation, both of which are affected in TS.^37, 38^ In addition to motor control, speech production is often implicated in TS through the development of vocal tics. Speech production requires the precise control of many muscles, and this complex process requires coordination of activity in multiple brain regions to plan, sequence, time, execute, and monitor these movements, achieved by basal ganglia regulation of thalamocortical outputs to prefrontal cortical areas.^39^ Studies in disorders related to speech production have suggested that the basal ganglia, thalamus, and cerebellar-cortical and cortico-striato-pallidal thalamic connections might participate in the monitoring of planning, coordination, timing, sequencing, and selection of the appropriate motor programs during verbal production.^40^ Patients with damage to basal ganglia nuclei are commonly reported to have disturbances affecting speech production, and lesions of the striatum specifically (particularly the putamen) and pallidum have been linked to reduced speech output and initiation and poor articulatory and phonatory control.^39^

Additionally, previous findings suggest greater activation of the ventral striatum during positive emotion regulation,^41^ as well as enhanced functional connectivity between the ventral striatum and frontoparietal attentional regions during processing of motivationally salient reward cues.^42^ The thalamus also plays roles outside of motor control; the anterior paraventricular nucleus of the thalamus (PVT) can promote arousal in response to novel stimuli.^43^ It is also involved in responding to rewarding stimuli as well as affecting behavior reflecting reward. The posterior PVT is especially responsive to stressors and generally promotes – but can also inhibit – anxiety-like behavior. This understanding may relate to the shape and volume differences we found in basal ganglia structures and the pallidum’s association with observed changes in tic symptoms.

#### Thalamus.

Thalamus volume did not differ significantly between groups. When analyzing surfaces, however, we found that the thalamus displayed localized volume increases in NT children when compared to both TS children and healthy controls. Previous volume and surface morphology studies have found greater gray matter volume or outward deformation in the thalamus of TS children compared to healthy controls, which is again seen here in our surface comparison between TS and healthy children.^13, 41^ Further, we found a significant correlation between a larger thalamus at baseline and the subsequent lesser improvement in tic symptoms. The position of the thalamus in CSTC circuits, mediating output from motor-related basal ganglia and motor areas of the cerebral cortex, supports its involvement in motor control.^42^ Outward surface deformation or increased volume in the medial thalamus may reflect a pathological process that leads to greater persistence of tics. As one possible mechanism, pruning of local synaptic connections in healthy development reduces the risk of tic persistence, and deficiencies in that pruning help reinforce tics.

The involvement of these subcortical structures in various neurological pathologies raises the question of what distinguishes the patterns observed in chronic tic development. One possibility lies in additional vulnerabilities, such as the genetic predispositions illuminated in a study conducted by Qi and colleagues to explore the genetic underpinnings of tic disorders.^43^ Perhaps the interplay between these genetic factors and concurrent neurodevelopmental abnormalities contributes to the manifestation of tics.

Our results are also consistent with the results of a meta-analysis on task-based fMRI studies in patients with TS.^44^ In the thalamus, the meta-analysis showed a clear overlap of all the conditions involving various aspects of voluntary motor execution, response inhibition, and tic generation. The pallidum and thalamus both showed more consistent activation in free-to-tic conditions. The meta-analysis also identified a positive correlation between tic severity and BOLD activity in the thalamus and putamen.^44^ Further, another study using whole brain diffusion tensor imaging (DTI) found alterations in the thalamus and putamen, congruent with other DTI studies in adult TS patients.^45^ Similarly, in the putamen and thalamus, we found that the NT group exhibited localized volume gain compared to the TS group, and the TS group showed localized volume gain compared to the healthy controls. It seems that these regions are implicated in both tic severity and persistence. In the thalamus, most of the patterns of outward deformation seen in the NT and TS comparisons with healthy controls (Figures 2F and 3E) were specifically found in the centromedian thalamus. This region in the thalamus has become one of the most often targeted regions for deep brain stimulation in patients with treatment-resistant tic disorders, as an excitatory feedback loop exists from the thalamus to the striatum originating in the centromedianparafasicular nuclei complex (CMPf) of midline thalamic nuclei.^46^ An elevated thalamocortical drive due to excessive activation of striatal neurons is thought to contribute to tic-related motor patterns in TS.

Further, when considering a volume increase–decrease model, we can perhaps consider the NT and TS groups to be different points on the same timeline. In this case, the strongest group differences indicated by larger subcortical volumes and outward deformation are seen in NT children when compared to TS and HC groups, and these results either normalize or reverse as the tics persist or disappear over time. A similar pattern was found when studying children and adults with autism spectrum disorder (ASD) compared with healthy participants. Compared with control participants, patients with ASD showed the strongest group differences (increased thickness in the frontal areas and decreased thickness in the temporal lobe) during childhood and adolescence, with normalized or even reversed thickness results in adulthood.^47^ These results suggested a complex developmental course for frontal, temporal, and subcortical structures in ASD. When comparing children and adults with TS, hippocampus and amygdala volumes declined significantly with age in the TS group but not in controls.^34^ In the case of TS, the subcortical enlargement seen in the NT group could possibly delay the onset of typical developmental processes, and then subsequent normalization occurs, such that chronic cases (TS/CTD) exhibit less abnormality than those with newly developed tics when compared to healthy controls.

An unexpected outcome of this study is that the NT-HC or NT-TS deformation values did not significantly predict 12-month TTS. We postulate that this outcome may be the result of basing our prediction on these two assumptions: 1) when the brain at a baseline point exhibits structural deficits, symptoms at a later point in time will worsen. This assumption is based on trends of known conditions, such as dementia and other neurological disorders, in which functional deterioration follows structural deficits.^48^ The other assumption was that 2) the deficits will continue to worsen, followed by worsening symptom outcomes. However, our results suggest that potentially neither of these assumptions were born out. Another explanation is that brain plasticity could be occurring.^49^ Thus, NT and TS participants may be learning to compensate to maintain behavior despite the neuroanatomical changes,^50^ so that behavioral changes (worsening tic symptoms) did not follow. Further, it is also possible that the brain structural deficits seen at baseline did not actually worsen. In order to investigate this latter claim, longitudinal analyses need to be conducted to examine neuroanatomical changes at a later point, as well as to track improvement or worsening of tic symptoms.

Thus, a limitation of this project is that we currently lack 12-month clinical data for a fraction of the NT participants for whom we have baseline scan data, so we were somewhat limited in which participants we could include in predictive analyses. Additionally, our healthy controls were collected across different studies/sources. While we performed harmonization steps (such as adjusting for voxel size differences), they were still not all from the same cohort and thus had slightly varying scanning parameters. We had also planned in advance to correct for differences in TIV, and the results showing significantly smaller TIV in the NT group confirmed the need for that plan. However, if all regional volumes in the NT group differ in the same direction from the other groups, one wonders whether the apparent regional changes simply reflect the whole-brain difference. However, even without correction for TIV, the hippocampal volume is still numerically larger in the NT group, taking age and sex into account. It is premature to draw conclusions regarding the pathophysiological implications of our findings without access to a larger set of longitudinal data. Cross-sectional data does not offer insights into underlying mechanisms and restricts a comprehensive exploration of the neuropsychological processes over time, thus highlighting the necessity for longitudinal analyses to strengthen predictive analyses.

Another concept to note is that NT and TS/CTD may exist on a spectrum rather than being discrete diagnoses arbitrarily based on symptom duration.^51^ This emerging view could potentially shape our future direction, such as shifting focus towards more dimensional analyses rather than categorical comparisons between groups. A larger study could similarly investigate differences between patients with CTD versus TS.

Primarily, we aimed to gain insight into the pathophysiology of tics, especially by investigating early in the development of the disorder to allow confident conclusions about causation. The work, however, may also have clinical relevance for precision medicine. Most patients and their parents request additional prognostic information, and including baseline subcortical shape data in the clinical features of an individual may improve the prediction of outcome. Additionally, some children have very severe tics that greatly impair their lives and that respond inadequately to behavioral and pharmacological therapies. In these cases, deep brain stimulation can be recommended, and pathophysiological research is needed to help identify potentially effective targets.

## Conclusion

Nevertheless, the New Tics study does provide the first imaging results from Provisional Tic Disorder. These new findings have potential clinical relevance, such as strengthening the possibility to identify ideal targets for treatment optimization. Continuing to investigate neuroanatomical characteristics in Provisional Tic Disorder may also further provide insight into prognostic biomarkers. In many children with Provisional Tic Disorder, tics improve within the first year, often to the point of clinical insignificance, but previously there was a paucity of information to predict which children would instead go on to have more severe tics over time. Understanding the mechanisms related to these outcomes may provide clinical insight into the pathophysiological traits in this complex disease and thus potentially guide interventions such as conventional or adaptative deep brain stimulation in drug-resistant cases, leading to an individualized treatment. A recent meta-analysis reports that deep brain stimulation in TS leads to a 40% improvement on tic severity scales on average.^52^ However, given the invasiveness of deep brain stimulation, predictors of prognosis are urgently needed to direct deep brain stimulation only to those children least likely to improve spontaneously. Finally, with a deeper understanding of neural networks related to the recent development of tics and progression of symptoms, we may be able to provide more ideal targets that could be manipulated to prevent the worsening of tic symptoms.

## Figures and Tables

**Table 1.** Participant Characteristics at Baseline or 12-Months (as specified under “Sample Characteristic”). Values indicate number or mean ± SD unless indicated otherwise. Sex, mean age (all near 8 years old), scanner type used, and race did not differ significantly between groups

Sample Characteristic	HC	NT	TS	Statistics
Total N (M/F)	60 (44 M/16F)	88 (63 M/25F)	39 (28 M/11F)	*χ*^2^ = 0.06, *p* = *0.97*
Mean age (SD) at baseline (years)	8.3 ± 2.0	7.8 ± 1.9	8.4 ± 1.9	F = 1.83, *p* = *0.16*
Scanner Type	15 Trio/45 Prisma	28 Trio/60 Prisma	8 Trio/31 Prisma	*χ*^2^ = 1.97, *p* = *0.37*
Race	44 White/10 Black or African American/5 More than one race/1 Unknown or not reported	73 White/6 Black or African American/6 More than one race/1 Asian/2 Unknown or not reported	30 White/8 More than one race/1 Asian	*χ*^2^ = 17.10, *p* = *0.07*
Mean TTS at baseline	N/A	16.96 ± 5.64; *n = 80*	20.79 ± 7.22; *n = 39*	N/A
Mean TTS at 12 months	N/A	14.16 ± 6.94; *n* = 80	N/A	N/A

**Table 2.** Subcortical Structural Volumes, Accounting for Age and Sex. Significant group differences were found in the hippocampus using a one-way ANCOVA while controlling for age and sex. Post hoc Tukey HSD tests further revealed a specific group difference

Structure	HC	NT	TS	ANCOVA	Cohen’s d
Hippocampus	5394 ± 168	5796 ± 140	5477 ± 205	F(2,182) = 2.81, p = 0.06	d _NT-HC_ = 0.31 d _TS-HC_ = 0.06 d _NT-TS_ = 0.25
*L Hippocampus*	2487 ± 77	2643 ± 64	2507 ± 94	F(2,182) = 2.14, p = 0.12	d _NT-HC_ = 0.26 d _TS-HC_ = 0.03 d _NT-TS_ = 0.23
^1^ *R Hippocampus*	2907 ± 92	3153 ± 77	2970 ± 112	F(2,182) = 3.38, p = 0.04	d _NT-HC_ = 0.34 d _TS-HC_ = 0.09 d _NT-TS_ = 0.26
Amygdala	2782 ± 88	2943 ± 73	2768 ± 107	F(2,182) = 2.01, p = 0.14	d _NT-HC_ = 0.24 d _TS-HC_ = −0.02 d _NT-TS_ = 0.26
*L Amygdala*	1359 ± 43	1425 ± 39	1348 ± 52	F(2,182) = 1.51, p = 0.22	d _NT-HC_ = 0.19 d _TS-HC_ = −0.03 d _NT-TS_ = 0.22
*R Amygdala*	1423 ± 46	1518 ± 38	1420 ± 56	F(2,182) = 2.45, p = 0.09	d _NT-HC_ = 0.27 d _TS-HC_ = −0.01 d _NT-TS_ = 0.28
Caudate	7671 ± 274	7700 ± 228	7201 ± 334	F(2,182) = 0.83, p = 0.44	d _NT-HC_ = 0.01 d _TS-HC_ = −0.22 d _NT-TS_ = 0.24
*L Caudate*	3784 ± 134	3796 ± 112	3568 ± 164	F(2,182) = 0.72, p = 0.49	d _NT-HC_ = 0.01 d _TS-HC_ = −0.21 d _NT-TS_ = 0.22
*R Caudate*	3887 ± 140	3904 ± 117	3633 ± 171	F(2,182) = 0.94, p = 0.39	d _NT-HC_ = 0.02 d _TS-HC_ = −0.24 d _NT-TS_ = 0.25
Accumbens	877 ± 32	878 ± 27	831 ± 39	F(2,182) = 0.50, p = 0.61	d _NT-HC_ = 0.00 d _TS-HC_ = −0.19 d _NT-TS_ = 0.19
*L Accumbens*	438 ± 16	439 ± 14	418 ± 20	F(2,182) = 0.36, p = 0.70	d _NT-HC_ = 0.01 d _TS-HC_ = −0.16 d _NT-TS_ = 0.16
*R Accumbens*	439 ± 16	439 ± 13	413 ± 19	F(2,182) = 0.65, p = 0.53	d _NT-HC_ = 0.00 d _TS-HC_ = −0.21 d _NT-TS_ = 0.22
Putamen	10 766 ± 385	10 592 ± 321	10 308 ± 470	F(2,182) = 0.29, p = 0.75	d _NT-HC_ = −0.06 d _TS-HC_ = −0.15 d NT-TS = 0.10
*L Putamen*	5214 ± 187	5142 ± 156	4986 ± 228	F(2,182) = 0.31, p = 0.74	d _NT-HC_ = −0.05 d _TS-HC_ = −0.16 d _NT-TS_ = 0.11
*R Putamen*	5552 ± 198	5451 ± 165	5322 ± 242	F(2,182) = 0.28, p = 0.76	d _NT-HC_ = −0.07 d _TS-HC_ = −0.15 d _NT-TS_ = 0.08
Pallidum	3677 ± 122	3616 ± 102	3624 ± 149	F(2,182) = 0.09, p = 0.91	d _NT-HC_ = −0.06 d _TS-HC_ = −0.06 d _NT-TS_ = −0.01
*L Pallidum*	1836 ± 61	1806 ± 51	1813 ± 75	F(2,182) = 0.08, p = 0.92	d _NT-HC_ = −0.06 d _TS-HC_ = −0.05 d _NT-TS_ = −0.01
*R Pallidum*	1841 ± 61	1810 ± 51	1811 ± 75	F(2,182) = 0.10, p = 0.91	d _NT-HC_ = −0.07 d _TS-HC_ = −0.06 d _NT-TS_ = 0.00
Thalamus	14 868 ± 521	14 438 ± 434	14 083 ± 635	F(2,182) = 0.50, p = 0.61	d _NT-HC_ = −0.11 d _TS-HC_ = −0.20 d _NT-TS_ = 0.09
*L Thalamus*	7558 ± 263	7358 ± 219	7165 ± 321	F(2,182) = 0.48, p = 0.62	d _NT-HC_ = −0.10 d _TS-HC_ = −0.20 d _NT-TS_ = 0.09
*R Thalamus*	7310 ± 258	7080 ± 215	6918 ± 315	F(2,182) = 0.52, p = 0.60	d _NT-HC_ = −0.10 d _TS-HC_ = −0.20 d _NT-TS_ = 0.08
Hippocampus (vNavs)	5050 ± 296	5278 ± 223	5250 ± 287	F(2,102) = 3.08, p = 0.05	d _NT-HC_ = 0.87 d _TS-HC_ = 0.69 d _NT-TS_ = 0.11

1 NT had larger right hippocampal volumes than healthy controls (p = 0.04).

Values indicate estimated marginal means ± SE unless indicated otherwise.

**Table 3.** Partial Correlation between Baseline Volume and TTS (controlled for screen TTS, age, & sex; n = 80)

Structure	TTS Change (r)	p-Value
Hippocampus	0.18	0.12
Amygdala	0.17	0.14
Caudate	0.21	0.06
Accumbens	0.15	0.20
Putamen	0.19	0.09
Pallidum	0.24	0.04
Thalamus	0.23	0.05
TIV	−0.14	0.23

**Table 4.** Group Comparisons of Deformation within TS-HC Significant Vertices. Significant group differences were found in the caudate using a one-way ANCOVA while controlling for age and sex. Post hoc Tukey HSD tests further revealed a specific group difference

Group Comparisons of Deformation within TS-HC Significant Vertices *n = 187*					
Structure	HC (mm)	NT (mm)	TS (mm)	ANCOVA	TukeyHSD
Caudate	−0.0489	−0.0510	0.1903	F(2,182) = 4.39, p = 0.01	^1^ TS-HC: p = 0.03 ^2^ TS-NT: p = 0.02
Accumbens	−0.0611	0.0066	0.0792	F(2,182) =1.91, p = 0.15	N/A
Putamen	−0.0222	−0.0158	0.0698	F(2,182) = 0.82, p = 0.44	N/A
Thalamus	−0.0350	−0.0039	0.0627	F(2,182) = 0.45, p = 0.64	N/A
All significant vertices	−0.0465	−0.0116	0.0977	F(2,182) = 1.85, p = 0.16	N/A

1 In the caudate, TS demonstrated overall outward deformation from the population average in significant vertices compared to the overall inward deformation seen in healthy controls (p = 0.03).

2 In the caudate, TS also demonstrated overall outward deformation from the population average in significant vertices compared to the overall inward deformation seen in NT (p = 0.02). Values indicate mean deformation value unless indicated otherwise.
